# Successful Long-Term Use of Isavuconazole in a Tacrolimus-Treated Japanese Kidney Transplant Recipient With Disseminated Cryptococcosis and Probable Invasive Aspergillosis

**DOI:** 10.7759/cureus.103470

**Published:** 2026-02-12

**Authors:** Yoshihiko Akagawa, Nanaka Egawa, Toshihiro Shimizu, Yasuhisa Shinkai, Shinpei Ono, Shoji Koga, Kazuhiro Ishikawa

**Affiliations:** 1 Infection Control, Edogawa Hospital, Edogawa, JPN; 2 Urology, Edogawa Hospital, Edogawa, JPN; 3 Neurology, Edogawa Hospital, Edogawa, JPN; 4 Plastic, Reconstructive and Aesthetic Surgery, Nippon Medical School, Bunkyo, JPN

**Keywords:** cryptococcus neoformans infection, infectious diseases, infectious pneumonia, invasive aspergillosis (ia), isavuconazonium, kidney transplant recipient, meningitis

## Abstract

Disseminated cryptococcosis, particularly cryptococcal meningitis, remains a severe fungal infection with high mortality in immunocompromised patients and usually requires prolonged azole therapy. In kidney transplant recipients receiving tacrolimus (TAC), careful management of drug-drug interactions via CYP3A is essential to maintain stable blood concentrations. We report the case of a 44-year-old man with a history of focal segmental glomerulosclerosis who had undergone a living-donor kidney transplant three years earlier. He was receiving immunosuppressive therapy with TAC, mycophenolate mofetil, and methylprednisolone. The patient developed cryptococcal meningitis and skin and soft tissue infection due to *Cryptococcus neoformans*, together with probable invasive aspergillosis of the paranasal sinuses. He received induction therapy with liposomal amphotericin B and flucytosine for approximately 10 weeks, but persistent intracranial hypertension required placement of a ventriculoperitoneal shunt. Consolidation and maintenance therapy with isavuconazole (ISCZ) was then initiated and has been continued successfully for more than 12 months, without adverse events and with stable TAC concentrations. At the time of writing, the treatment duration has exceeded 365 days, and he remains under outpatient management with no signs of recurrence. To our knowledge, reports describing more than 12 months of ISCZ therapy for cryptococcal infection are limited. This case highlights its potential as a maintenance option in transplant recipients, particularly when fluconazole or voriconazole are not suitable due to drug-drug interactions with TAC.

## Introduction

Cryptococcosis is a severe opportunistic infection primarily caused by *Cryptococcus neoformans* or *Cryptococcus gattii* and is particularly significant in immunocompromised patients. While approximately 89% of cases occur in people living with human immunodeficiency virus (HIV) (PLHIV) [1], it is also observed in 2.8% of solid organ transplant (SOT) recipients [2]. Among these, cryptococcal meningitis is associated with a high mortality rate. The standard treatment for cryptococcal meningitis consists of an induction phase with liposomal amphotericin B (L-AMB) at 3-4 mg/kg daily and flucytosine (5-FC) at 25 mg/kg four times daily, followed by a consolidation phase with fluconazole (FLCZ) 400-800 mg daily for eight weeks, and then a maintenance phase with FLCZ 200 mg daily for 12 months, requiring prolonged therapy. When FLCZ cannot be used during the consolidation phase, voriconazole (VRCZ), posaconazole (PSCZ), and isavuconazole (ISCZ) are suggested as alternative agents [3]; however, formal clinical trials are lacking. To our knowledge, there are limited reports on the long-term use (≥12 months) of ISCZ for cryptococcal meningitis.

Invasive aspergillosis is another life-threatening fungal infection in immunocompromised individuals, including SOT recipients, and is generally treated with azole antifungals, such as VRCZ or PSCZ [3,4].

Tacrolimus (TAC) is widely used as an immunosuppressant in kidney transplant patients, but its cytochrome P450 3A (CYP3A)-mediated metabolism requires careful consideration of drug-drug interactions with azole antifungals. Azole antifungal agents inhibit CYP3A, but the strength of inhibition varies among individual agents. VRCZ and PSCZ are classified as having severe interactions, requiring a two-thirds reduction in TAC dosage, whereas ISCZ is considered to have a moderate interaction, with no dose reduction to approximately a 50% reduction suggested [5]. ISCZ is an antifungal agent with good central nervous system penetration that has demonstrated efficacy against invasive aspergillosis and mucormycosis. Given that long-term therapy of more than 12 months was anticipated in this case, ISCZ was selected because it allows for easier TAC dose adjustment.

This case report describes a Japanese kidney transplant patient with cryptococcal meningitis and probable invasive aspergillosis who was successfully treated with ISCZ. During this period, TAC trough levels remained stable without requiring complex dose adjustments. To our knowledge, this case also demonstrates the long-term (use for more than 12 months) safety of ISCZ use, although it should be noted that ISCZ is not a standard therapy for cryptococcal infection.

## Case presentation

A 44-year-old Japanese male presented to the outpatient clinic with a right lower leg ulcer that had appeared two weeks prior to admission, accompanied by loss of appetite and general malaise. His past medical history included focal segmental glomerulosclerosis, a living-donor kidney transplant three years earlier, and he also had hyperglycemia. He was receiving immunosuppressive therapy with tacrolimus (4 mg/day), mycophenolate mofetil (500 mg/day), and methylprednisolone (12 mg/day). In addition, he was on prophylaxis with valganciclovir (500 mg twice weekly) and trimethoprim/sulfamethoxazole (80 mg of trimethoprim twice weekly).

**Figure 1:**
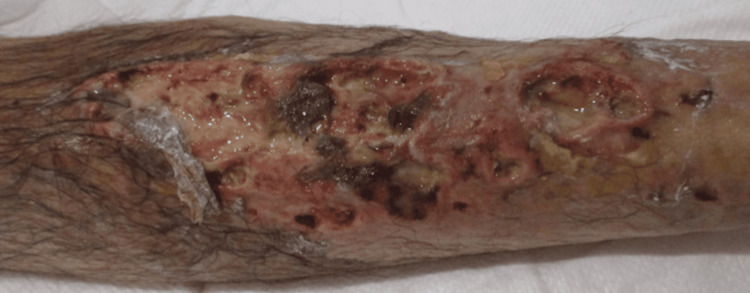
Right lower leg ulcer The skin ulcer was painful without accompanying heat or swelling.

On admission (hospital day one), he was alert and oriented. His vital signs were stable: temperature of 36.6 °C, blood pressure of 131/83 mmHg, pulse of 106 beats/min, and oxygen saturation of 98% on room air. Physical examination revealed a right lower leg ulcer (Figure 1).

Laboratory tests showed a C-reactive protein (CRP) level of 4.55 mg/dL. Serum creatinine was 2.08 mg/dL, and the estimated glomerular filtration rate (eGFR) was 29.0 mL/min/1.73 m². Liver function tests were within the normal range. Blood glucose was 408 mg/dL, and the tacrolimus trough level was 24.0 ng/mL. Serum β-D-glucan was 50.1 pg/mL, and the *Aspergillus *galactomannan antigen index was 1.0 (Table 1).

**Table 1:** Laboratory values on the first day of hospitalization

Laboratory test	Value	Reference range
Estimated glomerular filtration rate	29 mL/min/1.73 m²	≥ 60 mL/min/1.73 m²
Serum creatinine	2.08 mg/dL	0.33–1.17 mg/dL
Blood urea nitrogen	44 mg/dL	≤ 18.4 mg/dL
C-reactive protein	4.55 mg/dL	≤ 0.3 mg/dL
Serum albumin	2.7 g/dL	3.3–5.3 g/dL
Total bilirubin	0.64 mg/dL	0.2–1.0 mg/dL
Aspartate aminotransferase	17 U/L	11–35 U/L
Alanine aminotransferase	19 U/L	5–40 U/L
Alkaline phosphatase	82 U/L	38–113 U/L
Gamma-glutamyl transferase	31 U/L	8–75 U/L
Lactate dehydrogenase	542 U/L	124–222 U/L
Blood glucose	403 mg/dL	70–110 mg/dL
Tacrolimus trough concentration	24.0 ng/mL	5 ng/mL
Serum (1→3)-β-D-glucan	50.1 pg/mL	≤ 20 pg/mL
Serum Aspergillus galactomannan antigen	1.0 (index)	< 0.5
Cytomegalovirus pp65 antigenemia assay (C7-HRP)	Not detected	Not detected
Pneumocystis jirovecii polymerase chain reaction	Not detected	Not detected

**Figure 2:**
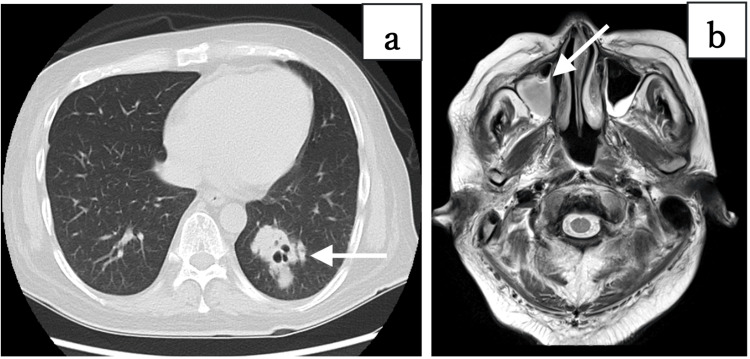
(a) A well-defined cavitary nodule was observed in the left middle lobe, as indicated by the white arrow on the chest CT. (b) The head MRI scan revealed fluid accumulation in the right maxillary sinus, as indicated by the white arrow on the T2-weighted image CT: computed tomography; MRI: magnetic resonance imaging

Chest computed tomography (CT) revealed a well-demarcated cavitary nodule in the left middle lobe (Figure 2a), and a head magnetic resonance imaging (MRI) demonstrated fluid retention in the right maxillary sinus (Figure 2b).

Gram staining of the leg ulcer revealed Gram-positive cocci and Gram-negative rods. Empiric treatment with meropenem 1 g every 24 hours was initiated but later changed to levofloxacin 500 mg every 24 hours after *Pseudomonas* species, *Clostridium perfringens*, and α-hemolytic *Streptococcus* were identified.

A yeast-like fungus was detected in the blood culture, and micafungin 100 mg every 24 hours was started on hospital day three. On hospital day seven, the patient lost consciousness, and a lumbar puncture was performed. The cerebrospinal fluid (CSF) was clear and colorless, with a cell count of 2/μL and a protein level of 28.7 mg/dL. However, the CSF glucose/blood glucose ratio was low at 0.24, and the CSF opening pressure was elevated at 20 cmH_2_O (Table 2). The elevated cerebrospinal fluid pressure was considered to reflect increased intracranial pressure, which was thought to be responsible for loss of consciousness.

**Table 2:** Cerebrospinal fluid examination findings on the seventh day of hospitalization

Test	Value	Reference range
Blood glucose	362 mg/dL	70–110 mg/dL
CSF appearance	Clear and colorless	Clear and colorless
CSF cell count	2 cells/mm³	≤ 5 cells/mm³
CSF protein concentration	28.7 mg/dL	10–50 mg/dL
CSF glucose concentration	88 mg/dL	50–80 mg/dL
CSF glucose/blood glucose ratio	0.24	≥ 0.6
CSF opening pressure	20 cmH_2_O	7-18 cmH_2_O

**Figure 3:**
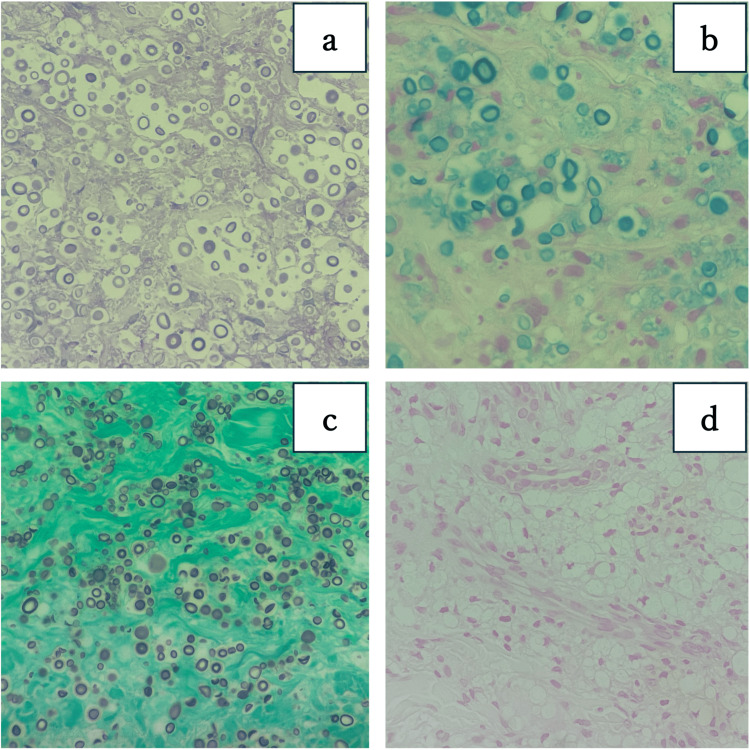
The skin biopsy pathology results It were positive for (a) periodic acid-Schiff, (b) Alcian blue stain, and (c) Grocott methenamine silver staining and negative for (d) Fontana Masson stain.

The skin biopsy pathology results were positive for periodic acid-Schiff, Alcian blue stain, and Grocott methenamine silver staining, and negative for Fontana Masson stain, and a capsulated yeast-like fungus was identified as *C. neoformans* (Figure 3).

**Figure 4:**
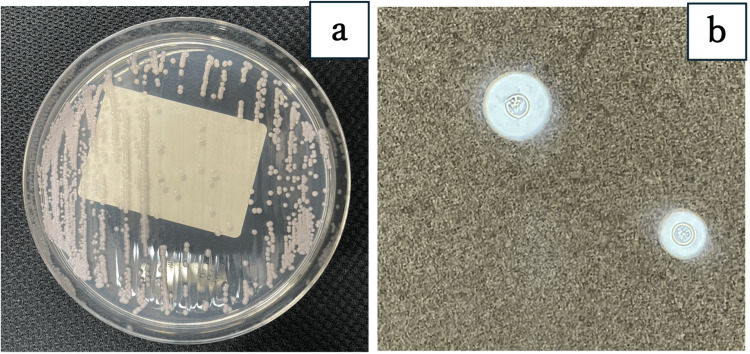
Cryptococcus neoformans colony and India ink stain (a) The culture on CHROMagar Candida medium formed a white, mucoid colony. (b) India ink staining of a CSF specimen revealed a capsulated yeast-like fungus.

Blood and CSF cultures on CHROMagar Candida medium (Kanto Chemical Co., Tokyo, Japan) formed white, mucoid colonies (Figure 4a), and India ink staining yielded positive results (Figure 4b). The isolate was further identified as *C. neoformans* by matrix-assisted laser desorption/ionization (MALDI) Biotyper (Bruker Corporation, Billerica, MA). The minimum inhibitory concentration (MIC) values were determined according to the Clinical and Laboratory Standards Institute-approved standard, M27-A3 [6], using yeast-like fungus DP (Eiken Chemical Co., Tokyo, Japan) and automatically analyzed by DPS-MIC192/ID system(Eiken Chemical Co., Tokyo, Japan) (Table 3).

**Table 3:** Susceptibility results for Cryptococcus neoformans MIC: minimum inhibitory concentration

Antifungal agent	MIC break point (μg/mL)	MIC (μg/mL)
Amphotericin B	No date	0.5
Flucytosine	No date	4
Miconazole	No date	0.25
Fluconazole	No date	2
Itraconazole	No date	0.12
Micafungin	No date	>16
Caspofungin	No date	16
Voriconazole	No date	0.12

**Figure 5:**
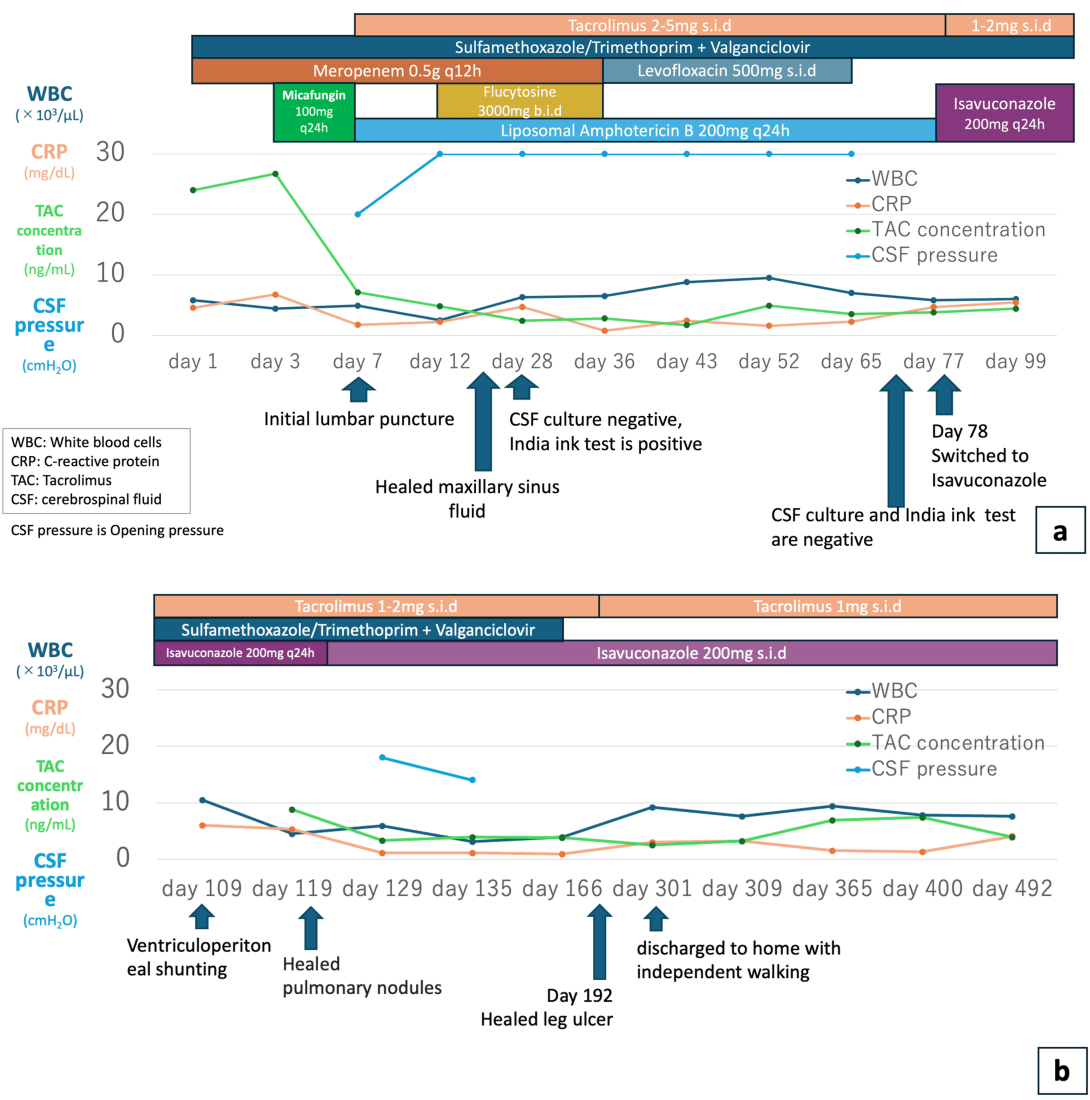
(a) Elevated cerebrospinal fluid pressure (blue line) persisted after initiation of induction therapy; therefore, after confirming conversion to negative India ink staining, treatment was switched to isavuconazole (ISCZ). (b) After CSF pressure was well controlled with ventriculoperitoneal (VP) shunt placement and improvement in consciousness was confirmed, isavuconazole was transitioned to oral administration (a) Clinical course from admission to shunt placement. Meropenem (MEPM) was started for the right lower leg ulcer, and micafungin (MCFG) was initiated after a yeast-like fungus was found in the blood culture. On day seven, L-AMB was started due to altered consciousness, and 5-FC was added on day 12. On day 77, after confirming negative cerebrospinal fluid (CSF) culture and India ink stain, the treatment was switched to intravenous isavuconazole (ISCZ). Tacrolimus (TAC) was reduced to 1-2 mg/day from the third day after ISCZ initiation, and blood concentrations were stable. (b) A VP shunt was placed on day 109, after which the CSF pressure stabilized and consciousness improved. On day 119, the lung nodule also disappeared, and ISCZ was switched to an oral formulation. The patient was discharged home on day 301, able to walk independently.

Based on these findings, the patient was diagnosed with disseminated cryptococcosis, involving meningitis, pneumonia, and skin infection, as well as probable invasive aspergillosis, involving pneumonia and sinusitis, according to the diagnostic criteria for invasive fungal disease (IFD) established by the European Organisation for Research and Treatment of Cancer/Mycoses Study Group (EORTC/MSG) [7]. Induction therapy was initiated with L-AMB 200 mg (5 mg/kg) once daily and 5-FC 1500 mg (25 mg/kg) every 12 hours, showing clinical course and laboratory values (Figure 5a). The blood cultures became negative on hospital day nine, and the CSF cultures became negative on hospital day 28, although the India ink stain remained positive. The maxillary sinus fluid collection resolved on hospital day 24. Despite these mycological responses, CSF pressure remained elevated and altered consciousness persisted; therefore, a ventriculoperitoneal (VP) shunt was planned. On hospital day 70, the CSF India ink stain also turned negative. On hospital day 78, the treatment was switched to ISCZ 200 mg once daily as consolidation therapy. On hospital day 109, a VP shunt was placed, and on hospital day 119, when CSF pressure stabilized, consciousness improved, and oral intake resumed. On the same day, the pulmonary nodule resolved, and ISCZ was switched to an oral formulation (Figure 5b). The right lower leg ulcer was completely healed on hospital day 192, and the patient was discharged home on hospital day 301, able to walk independently. After initiation of ISCZ, TAC was reduced to 1-2 mg/day. Following discharge, the TAC trough concentration remained stable with a maintenance dose of 1 mg once daily, without the need for further adjustment. At the time of writing, the treatment duration has exceeded 365 days, and he remains under outpatient follow-up with no signs of recurrence or adverse events, such as hepatic dysfunction, renal impairment, or QT interval prolongation, observed (Table 4).

**Table 4:** Laboratory data on day 492 of hospitalization

Laboratory test	Value	Reference range
Estimated glomerular filtration rate	42	≥ 60 mL/min/1.73 m²
Serum creatinine	1.47	0.33–1.17 mg/dL
Serum albumin	3.1	3.3–5.3 g/dL
Total bilirubin	0.47	0.2–1.0 mg/dL
Aspartate aminotransferase	25	11–35 U/L
Alanine aminotransferase	18	5–40 U/L
Blood glucose	104	70–110 mg/dL
Tacrolimus trough concentration	3.1	5 ng/mL
Blood urea nitrogen	15	≤ 18.4 mg/dL

**Figure 6:**
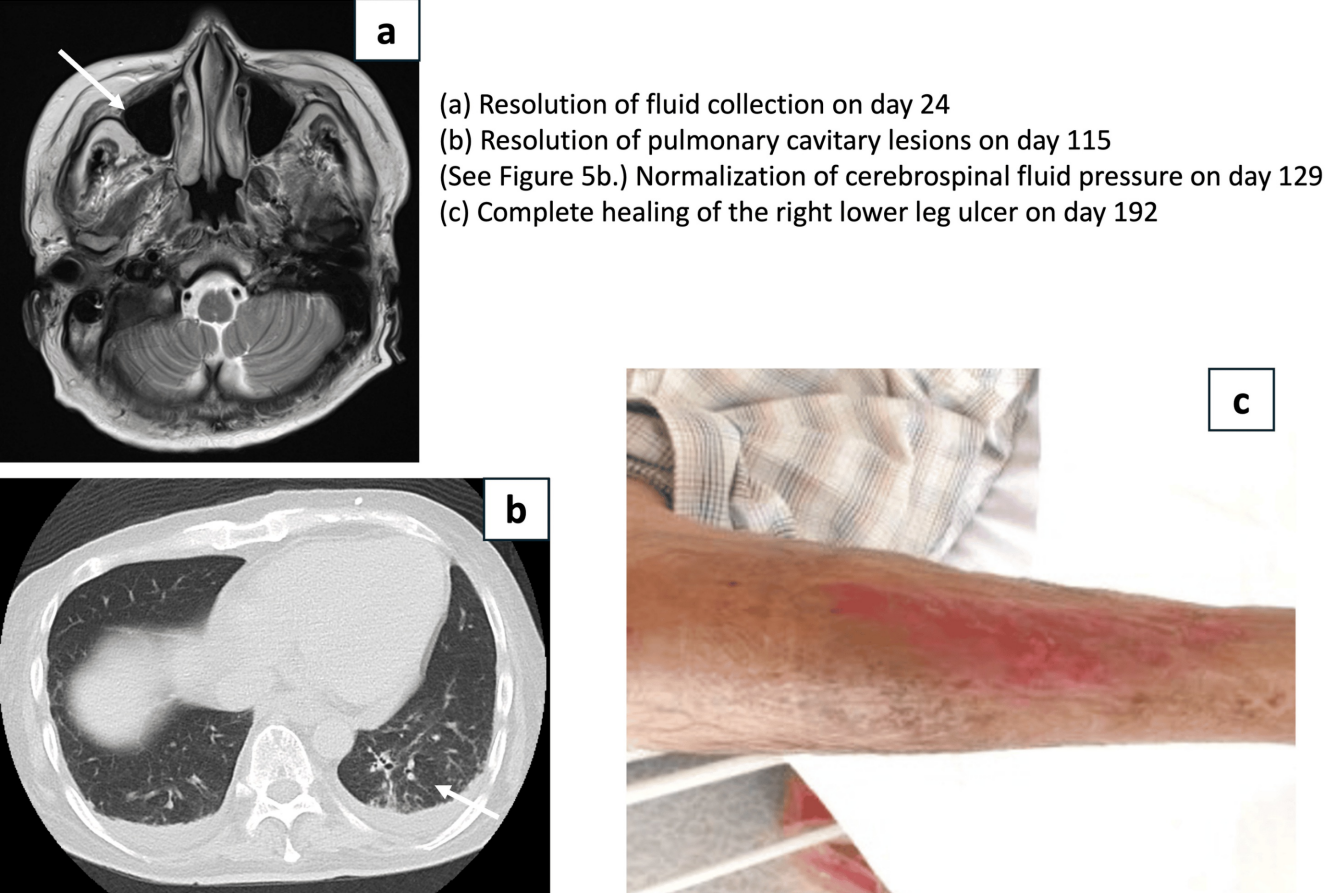
Time to improvement of each symptom (a) On the head MRI obtained on day 24, improvement of the fluid collection indicated by the white arrow was confirmed. (b) On the chest CT obtained on day 115, improvement of the cavitary nodular lesion in the left middle lobe, indicated by the white arrow, was confirmed. (c) On day 192, complete epithelialization of the entire ulcer of the right lower leg was observed. On day 129 after ventriculoperitoneal (VP) shunt placement, the cerebrospinal fluid pressure had normalized, and no further lumbar punctures were performed after day 135 (see Figure 5b).

Supplementary information: Although the fluid collection in the right maxillary sinus resolved rapidly, complete healing of the right lower leg ulcer required nearly 200 days (Figure 6).

In this case, the possibility of cryptococcal meningitis and invasive aspergillosis could not be ruled out. In a kidney transplant recipient in whom standard therapies such as FLCZ and VRCZ could not be used because of drug-drug interactions with TAC, ISCZ allowed treatment for more than 12 months without the need for complex TAC dose adjustments. However, the impaired consciousness was due to elevated intracranial pressure and did not improve with ISCZ alone; placement of a VP shunt enabled adequate control of cerebrospinal fluid pressure and led to improvement in consciousness.

ISCZ is being continued as secondary prophylaxis for invasive aspergillosis after 12 months of treatment.

## Discussion

This case involved a kidney transplant recipient on oral TAC who presented with disseminated cryptococcosis and a possible invasive aspergillosis. The choice of treatment drugs required consideration of potential drug interactions with TAC. This case highlights the potential efficacy and safety of ISCZ as an alternative to FLCZ when the latter cannot be used due to drug interactions or when aspergillosis cannot be excluded in a Japanese kidney transplant patient with disseminated cryptococcal meningitis. Most reported cases of cryptococcal infection occur in PLHIV patients; however, approximately 2.8% are observed in solid organ transplant recipients. Among these, the central nervous system is the most common site of infection (55%), followed by the lungs (6%) and skin, bone, or joint involvement (13%). The overall mortality rate is approximately 42% and is significantly higher in patients with central nervous system involvement (28.5%). In addition, TAC-based immunosuppressive therapy is associated with a lower incidence of central nervous system involvement and a higher likelihood of skin and soft tissue lesions compared with regimens not based on TAC [2].

The diagnosis of invasive aspergillosis is challenging. According to the EORTC/MSG criteria, this case was classified as probable invasive aspergillosis based on host factors, clinical features, and mycological evidence [8]. False-positive results for *Aspergillus* galactomannan antigen have been reported in cryptococcal infections [9]. In the present case, bronchoalveolar lavage and lung biopsy could not be performed due to the patient's impaired consciousness, and *Aspergillus *spp. could not be definitively identified. However, given the presence of host risk factors (kidney transplantation), clinical findings (pneumonia and sinusitis), and a positive galactomannan antigen index (1.0), a diagnosis of probable invasive aspergillosis was made.

Current guidelines for cryptococcal meningitis recommend FLCZ as first-line therapy [3], while those for invasive pulmonary aspergillosis recommend VRCZ [4]. In this case, the coexistence of disseminated cryptococcosis and probable invasive aspergillosis, combined with the need to minimize drug interactions with TAC, led to the use of ISCZ. Although the MIC for ISCZ against this isolate was not measured, ISCZ was considered appropriate based on previous reports showing MIC values against *C. neoformans* and *Aspergillus* spp. comparable to those of VRCZ [10]. Ideally, the MIC of ISCZ should have been measured; however, our laboratory equipment was not capable of performing this test. In addition, no breakpoints have been established by the Clinical and Laboratory Standards Institute, and we hope that these will be defined in the future.

The efficacy and safety of ISCZ in cryptococcal meningitis have not been evaluated in randomized controlled trials and are limited to case reports and case series, primarily in PLHIV, which generally suggest favorable outcomes [11-13]. All azole antifungal agents inhibit CYP3A and thereby affect blood concentrations of TAC, a CYP3A substrate; however, the degree of inhibition varies among agents. VRCZ and PSCZ are classified as having severe interactions, with package inserts recommending a two-thirds reduction in TAC dosage, whereas ISCZ is considered to have a moderate interaction and carries no specific dosing recommendations for TAC in the package insert [5]. Therefore, we considered that ISCZ would allow for easier TAC dose adjustment. Appropriate management of TAC blood concentrations is critical to prevent rejection of the transplanted kidney. In terms of safety, ISCZ has been associated with a lower incidence of hepatotoxicity and fewer treatment discontinuations due to adverse events compared with VRCZ, PSCZ, and amphotericin B [12]. Intravenous VRCZ contains sulfobutyl ether-β-cyclodextrin (SBECD), whereas ISCZ does not. Concomitant use with immunosuppressants is a strong predictor of renal impairment [14]. Treatment of cryptococcal meningitis requires long-term therapy (≥12 months), and continuous hospitalization for the entire course is impractical. In the outpatient setting, where frequent TAC monitoring is difficult, both the stability of TAC blood concentrations and the safety of azole antifungal agents are important considerations; therefore, ISCZ was selected in this case.

The duration of ISCZ administration was based on the cryptococcal meningitis guidelines [4], with maintenance therapy lasting 12 months. Treatment strategies for solid organ transplant recipients are often extrapolated from HIV data, and robust evidence in transplant populations is lacking. The longest administration period found in my research was 182 days [15]; however, in this case, the patient has been receiving the drug for over 365 days without any adverse events such as liver or kidney damage. We also considered the timing for discontinuing ISCZ, but decided to continue the administration as a form of secondary prevention for aspergillosis as long as immunosuppressive therapy is ongoing.

## Conclusions

This case of a Japanese kidney transplant recipient receiving oral tacrolimus who had a coinfection of *C. neoformans *meningitis and probable invasive aspergillosis. Based on the IFD definitions proposed by the EORTC/MSG, this case fulfilled the host (the patient was on oral tacrolimus as immunosuppressive therapy) and clinical (a well-circumscribed cavitary nodule and fluid retention in the maxillary sinus), and mycological criteria (serum *Aspergillus *galactomannan antigen and β-D-glucan were positive), which was classified as probable invasive aspergillosis. It should be noted that a definitive diagnosis of invasive aspergillosis could not be established.

The patient was successfully treated with ISCZ for over 365 days without any adverse events. This case suggests that ISCZ may be an effective and safe alternative to fluconazole for disseminated *C. neoformans *infection when drug interactions are a concern or invasive aspergillosis cannot be definitively excluded. However, the absence of directly determined ISCZ MIC values represents a limitation, and additional evidence is required to establish this therapeutic approach.
